# Head Stabilization in the Pigeon: Role of Vision to Correct for Translational and Rotational Disturbances

**DOI:** 10.3389/fnins.2017.00551

**Published:** 2017-10-05

**Authors:** Leslie M. Theunissen, Nikolaus F. Troje

**Affiliations:** ^1^Biomotion Lab, Department of Psychology, Department of Biology, School of Computing, Queen's University Kingston, Kingston, ON, Canada; ^2^Applied Cognitive Psychology, Faculty of Engineering, Computer Science and Psychology, Institute of Psychology and Education, Ulm University, Ulm, Germany

**Keywords:** motion capture, head stabilization, pigeon, motor control, sensory feedback, vestibular system, proprioception

## Abstract

Stabilization of the head in animals with limited capacity to move their eyes is key to maintain a stable image on the retina. In many birds, including pigeons, a prominent example for the important role of head stabilization is the characteristic head-bobbing behavior observed during walking. Multimodal sensory feedback from the eyes, the vestibular system and proprioceptors in body and neck is required to control head stabilization. Here, we trained unrestrained pigeons (*Columba livia*) to stand on a perch that was sinusoidally moved with a motion platform along all three translational and three rotational degrees of freedom. We varied the frequency of the perturbation and we recorded the pigeons' responses under both light and dark conditions. Head, body, and platform movements were assessed with a high-speed motion capture system and the data were used to compute gain and phase of head and body movements in response to the perturbations. Comparing responses under dark and light conditions, we estimated the contribution of visual feedback to the control of the head. Our results show that the head followed the movement of the motion platform to a large extent during translations, but it was almost perfectly stabilized against rotations. Visual feedback only improved head stabilization during translations but not during rotations. The body compensated rotations around the forward-backward and the lateral axis, but did not contribute to head stabilization during translations and rotations around the vertical axis. From the results, we conclude that head stabilization in response to translations and rotations depends on different sensory feedback and that visual feedback plays only a limited role for head stabilization during standing.

## Introduction

Vision is the most important sensory modality to obtain distant information about our environment. To ensure the perception of relevant information, animals do not only need specialized eyes (Jones et al., 2007), but also appropriate control mechanisms to coordinate the movements of their eyes in external space. This involves pursuit eye movements, exploratory saccades, but also eye and head movements to maintain fixation while compensating for body movements in space.

Humans can actively move their eyes in a wide range. They perform eye saccades to change fixation from one object to the other and smooth pursuit eye movements to keep moving objects in focus. To stabilize the eyes, two reflexes play major roles: the vestibulo-ocular reflex (VOR) and the optokinetic reflex (OKR, Angelaki and Cullen, 2008). The OKR, which is based on visual feedback, is especially helpful to fixate an object while there might be relative motion between the object and the observer. In contrast, the VOR can be seen as a feed-forward mechanism that uses linear and angular acceleration measured by the vestibular system to control eye muscles in order to stabilize the image on the retina.

Fixating by means of eye movements that compensate translations of the head and body through space achieves image stabilization only on the part of the retina that is fixating, generally the center of the fovea. Perifoveal regions will experience parallactic movements that increase with eccentricity and also depend on the distance of visual objects from the observer.

In humans, high acuity is concentrated in the relatively small area of the central fovea. Many vertebrates, particularly those with laterally places eyes and those under heavy predatory pressure distribute visual acuity over much larger areas of the visual field. This is particularly true for many birds (Jones et al., 2007). Pigeons, for instance, have two areas of high photoreceptor density in each eye and both of them are much larger than the four-degree fovea in the human eye (Land and Nilsson, 2012).

The only way to maintain a perfectly stable image over extended areas of the visual field is to not move the eye at all with respect to the visual environment—at least for short periods of time. Many birds adopt that strategy. A walking pigeon, for example, locks its head in space as long as possible and then thrusts it with one sudden, ballistic movement into a new position, where it again becomes motionless while the body catches up in a more continuous movement. The alternation of these so-called hold and thrust phases is called “head-bobbing” (Dunlap and Mowrer, 1930; Friedman, 1975; Frost, 1978; Troje and Frost, 2000). Head-bobbing occurs mainly during walking, but can also be observed in swimming birds.

While the saccadic head-movements during head-bobbing are mainly translatory, birds also rotate their head with sudden fast movements and then keep it relatively stable between these rotational thrusts (Kress et al., 2015). At least during locomotion on the ground and while perching, rotational head saccades are observed in all bird species. In contrast, the saccadic translational movements characteristic for head-bobbing are only observed in some bird species, but not in others.

Of course, there is a major difference between rotational and translational perturbations: for rotations, angular compensation of gaze must be always as large as the angular perturbation to ensure a stable image on the retina, whereas during translation required angular adjustments are generally small, particularly when fixating at distant objects (Land, 1999).

Given their physical nature, rotations, where retinal image velocity is perfectly predictable and does not depend on distance, and translations, where the velocity of the retinal flow depends on the distance to the observed object, require very different sensory systems to respond to the corresponding perturbations. In general, it is advantageous if the respective sensory system is located on the structure that is perturbed. For instance, the VOR in humans is so fast because the vestibular sensors are located on the head (the structure that is perturbed) rather than on the eye (the part that needs to be controlled). Direct measurements of the cause of the perturbation can be used for fast feed-forward control, while measurement of the structure that is controlled can only provide an error signal for slower feedback control (Wolpert et al., 1998).

The VOR in humans and other vertebrates clearly implements a feed-forward system that efficiently controls eye movements (Angelaki and Cullen, 2008). Likewise, in flies the halters located on the body are used for feed-forward control of head movements (Sandeman and Markl, 1980; Hengstenberg, 1988). However, it is not clear how birds control their head movements to maintain a stable image on the retina. Visual feedback alone is comparatively slow leading to blurred images, and if vestibular sensory information had to be used to stabilize position and orientation of the head (VCR), the sensors would move along with the structure that is meant to be controlled, i.e., the head (Wilson et al., 1994) and can therefore only be used for feedback control. In pigeons, a good candidate to provide direct information about body orientation is the lumbosacral vertebral canal system, which has been suggested to work similarly as the vestibular system as an equilibrium sense and might be able to measure rotational movements (Necker et al., 2000; Necker, 2005, 2006).

In pigeons, head-bobbing can be observed in response to visual flow (Friedman, 1975; Frost, 1978), which seems to be sufficient to elicit eye (optokinetic nystagmus, OKN, (Mowrer, 1936; Nye, 1969; Wallman and Velez, 1985) as well as head rotations (opto-collic reflex, OCR; (Gioanni, 1988a; Wallman and Letelier, 1993; Maurice et al., 2006). Head rotations based on the OCR account for about 80–90% of the gaze response if the head is free to move (Gioanni, 1988a). In addition, vestibular reflexes play a major role in gaze stabilization. The VOR—similar to humans but with smaller amplitudes—can induce rotational eye movements (Dickman and Angelaki, 1999; Dickman et al., 2000) and the vestibulo-collic reflex (VCR) serves head stabilization (Gioanni, 1988b; Goode et al., 1999). Although the VOR is under-compensatory in response to head rotations in the dark, gaze was well-stabilized in a head free condition (Haque and Dickman, 2005). Again, head movements contributed more to gaze compensation than eye movements. In a follow up study involving vestibular lesions, it was shown that the vestibular system plays a major role for gaze compensation (Haque et al., 2008). Furthermore, head stabilization is state dependent: When a pigeon is in a simulated flight mode, head stabilization in response to body rotations in darkness was much better compared to the non-flying condition (Maurice et al., 2006; McArthur and Dickman, 2011). These examples show that gaze is mainly stabilized through head stabilization in response to rotational perturbations.

Overall, optokinetic compensations of rotational perturbations can be found in almost any animal. Often they are not exclusively performed by eye movements but include head or even whole body movements, e.g., birds (Gioanni, 1988a), flies (Land, 1973) and even water flea (Frost, 1975). In contrast, translational perturbations are more often compensated with eye movements. The difference in the response to rotational and translational perturbations is particularly obvious in birds: as far as we know, all birds conduct head rotations by means of sudden saccadic movements, whereas only some birds perform head-bobbing.

Here, we compared head stabilization in response to translational and rotational perturbations in pigeons. In contrast to most previous studies, where restrained birds were rotated in the dark or head movement was analyzed in response to visual motion, we used freely standing pigeons to allow the whole body, including legs, trunk and head to respond to perturbations of the platform on which they were standing either in light or dark conditions. This setup models the natural situation when a pigeon perches on a moving branch. To investigate head and body stabilization during translations and rotations, we trained pigeons to stand on a perch, which was sinusoidally translated along and rotated around the three spatial dimensions with different frequencies and amplitudes.

## Materials and methods

### Subjects

For the experiment, we used 10 (7 male, 3 female) rock doves (*Columba livia*) that were originally obtained from pigeon breeders in Ontario, Canada (Limestone City Flyers) and kept at Queen's University under veterinary supervision. The birds were between 4 and 10 years old, weighed between 380 and 520 g and were housed in a separate aviary for the duration of the experiment. Food and water were available *ad libitum* and light was kept on a 12 h on/off cycle. All experiments conformed to the ethics requirements for animal research of the Canadian Council on Animal Care (CCAC) and were approved by the Queen's Animal Care Committee.

### Experimental setup

**Figure 1:**
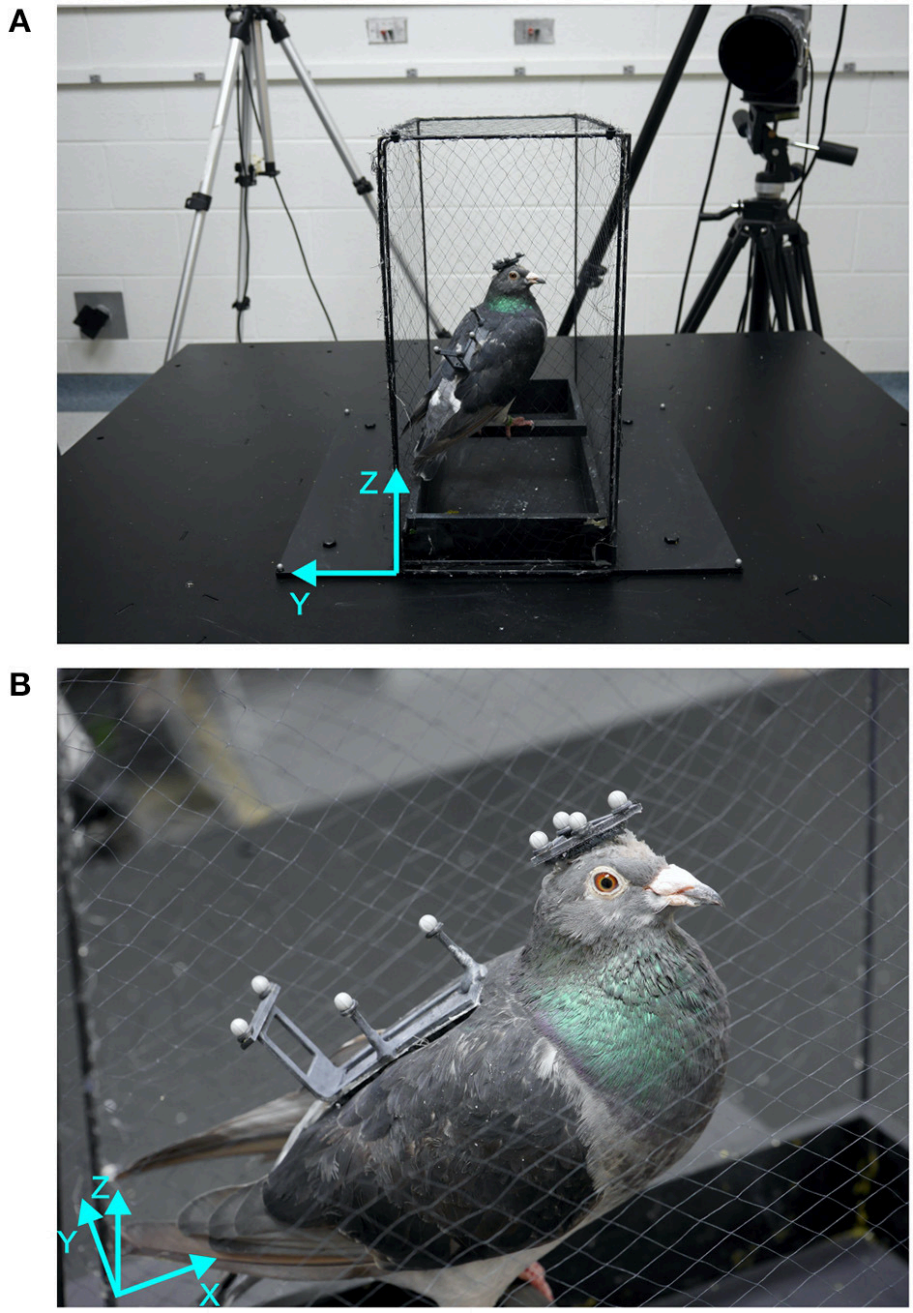
Setup and bird with markers. **(A)** The motion platform is shown with the custom built cage, one motion capture camera and a bird sitting on the perch aligned with the lateral axis of the platform. **(B)** Close-up of a bird standing on the perch with head and back marker patches attached with Velcro patches. The platform coordinate systems (cyan) show forward-backward (x), lateral (y), and vertical (z) axes.

The experimental setup consisted of a six degree of freedom motion platform (116 × 116 × 36 cm, W3s 6DOF Motion System, CKAS, Australia) onto which we had mounted a polyvinyl chloride box frame (41 × 20.5 × 41.5 cm) that was covered with mist netting (Figure 1A). The pigeons stood on a perch (19 × 1.5 × 1.5 cm) in the center of the frame. The perch was located 5 cm above the platform and was aligned with the lateral axis (y-axis) of the coordinate system used to describe and measure movements of motion platform, as well as head and body of the pigeon. Therefore, the coordinate system was defined as followed: The x-axis was the platform dimension along which the bird was perched (forward-backward). The y-axis was the horizontal axis perpendicular to x, which coincides with the lateral axis of the perched bird. The z-axis is the vertical axis (Figure 1).The platform was placed in the middle of a window-less room (3.2 × 3.4 m) in which the door, the cameras with tripods (Figure 1A), a table with metal boxes and a table with the measurement computer provided a rich visual environment during light conditions. In dark conditions, the room was completely darkened with every light source, e.g., power lights from the cameras and the computer, covered with tape and the computer monitor switched off. The only light in the room was the infrared light required by the optical motion capture system. The LEDs of that system are invisible to the human system and given that their light is entirely in the infrared part of the spectrum. The same is probably true for pigeons, because the absorption spectrum of the cone class in the pigeon retina with longest wavelength spectrum peaks at 565 nm, which is almost exactly where the human L-cone peaks (Bowmaker, 1977; Bowmaker et al., 1996). Movement of the motion platform was controlled with customized Python scripts that also synchronized the start of the motion capture recording via an analog signal sent to the motion capture system.

### Motion capture

The motion platform was surrounded by a five-camera motion capture system (Oqus 300 series, Qualisys AB, Sweden) that recorded three-dimensional positions of passive retro-reflective markers at a sample rate of 360 Hz with a spatial resolution (RMS error) smaller than 1 mm. Four markers were attached to the motion platform (Figure 1A) to record its movements with similar accuracy as the pigeon's movements.

To record the movements of the pigeons, we created two custom-made lightweight rigid frames, a triangle for the head (2 g) and a rectangle for the back (3 g, see Figure 1B). Each frame had four retro-reflective markers (3 × 3 Designs Ltd., 4 mm in diameter) attached to it. In order to attach the frames to the birds, two areas of feathers (one on the head and one on the back) were trimmed and patches of Velcro were attached to them using white crafts glue. These patches remained on the birds until data collection was finished. The frames with the motion capture markers were attached to the birds via the Velcro patches prior to an experimental session, and were removed once the session was completed.

### Perturbations

Perturbations consisted of sinusoidal movements of the motion platform with five different frequencies (0.25, 0.5, 1, 2, 4 Hz) for translations (x: forward-backward, y: lateral, z: vertical) and four different frequencies (0.25, 0.5, 1, 2 Hz) for rotations around x, y and z. By adjusting the amplitude of the movements to the different frequencies (Translations: 50, 25, 12.5, 6.25, 3.125 mm; Rotations: 9, 4.5, 2.25, 1.125 degrees) we kept the maximum velocity constant at 78.5 mm/s and 14 deg/s for translations and rotations, respectively. These amplitudes were chosen due to the limitations in amplitude (50 mm; 10 degrees), velocity (100 mm/s; 15 deg/s) and acceleration (2942 mm/s^2^; 150 deg/s^2^) that the motion platform was able to handle. The motion capture data revealed that the measured amplitudes, velocities and accelerations were close to the intended values for low frequencies, but a bit smaller for 2 and 4 Hz perturbations (Supplementary Table 1). The rotational acceleration limit (150 deg/s^2^) prevented us from rotating the platform with 4 Hz, because the maximum amplitude would have been unreasonably small (only 0.02 degrees).

### Procedure

The experimenter attached the two marker frames on the head and the back of the bird and placed the bird on the perch. The birds had been trained to stay on the perch and were not restrained. In the rare case that the bird stepped off the perch, the recoding was stopped and the whole session was repeated after the bird was placed back on the perch.

In total, we recorded 40 sessions (20 light and 20 dark) per bird within the same day. Light and dark conditions were blocked, such that five sessions, each containing perturbations in all six degrees of freedom (translations in and rotations around x, y and z) of the same frequency in light condition were followed by five sessions in dark condition. Sessions with low frequencies (0.25, 0.5, and 1 Hz) were recorded separately, whereas the two higher frequencies (2 and 4 Hz) were recorded together, to reduce the total duration of the experiment. Given that every direction was presented once during each session, sessions consisted of six (low frequencies) or nine (high-frequencies) trials (4 Hz only translations).

A trial was defined as perturbation along one direction and consisted of 10 sinusoidal cycles, except for trials of the highest frequency (4 Hz), which consisted of 20 cycles to provide 5 s of stimulation. This resulted in trial durations of 40, 20, 10, and 5 s, respectively. The order of directions was randomized and between directions, there was an interval of 2 s without perturbation.

### Data analysis

To analyse the movements of the platform, the head and the body, a rigid body model was assigned for each marker set (platform, head and body), marker trajectories were semi-automatically labeled and gaps were filled automatically using Qualisys QTM software. Position and orientation data of each rigid body model and marker data were exported to Matlab (The Mathworks, USA), where they were combined with data about timing and direction of each perturbation.

Before calculating gains and phases of the head with respect to the platform and body movement, we identified saccadic head movements. A saccadic head movement was identified when the difference between Euclidian head and body velocity was faster than three times the maximum velocity of the platform for at least nine consecutive frames (25 ms). Beginning and end of each saccade was then adjusted using a model that consisted of a concatenation of individual constant velocity movements and that was optimized with the simplex search method (Lagarias et al., 1998) that reduced the area between the original and the modeled data.

To calculate gain and phase, we first computed amplitudes and phases of platform, body and head in world coordinates with the help of the discrete Fourier transform:

(1) $$\begin{array}{r} X_{f}=\sum_{t}x_{q,t}e^{\;-i2\pi ft} \end{array}$$

Here, *x*_*q,t*_ denotes the position of *q* ∈ {*p* = *platform*, *h* = *head, b* = *body*} at time *t*, and *f* is the perturbation frequency. From this, we can compute amplitude *a* and phase φ of each motion:

(2) $$\begin{array}{r} a_{q}=\sqrt{ℜ{\left(X_{q}\right)}^{2}+ℑ{\left(X_{q}\right)}^{2}}\;and\;\varphi_{q}=\arctan\frac{ℑ\left(X_{q}\right)}{ℜ\left(X_{q}\right)} \end{array}$$

To quantify the head's response to platform perturbations, we first estimated the gain in world coordinates as $g^{w}=\frac{a_{h}}{a_{p}}$ and the time lag between head and platform motion as $\delta t=\frac{\varphi_{h}-\varphi_{p}}{\omega }$. Here, ω is the angular frequency ω = 2π*f*.

These estimates were then used to initialize a gradient decent optimization to fit the following model to the original data:

(3) $$\begin{array}{r} x_{h}\left(t\right)=g^{w}x_{p}\left(t-\delta t\right)+S\left(t\right) \end{array}$$

where *S*(*t*)denotes the saccade model that we described above. Initial values of *g*^*w*^ and δ*t* were adjusted with 300 iterations using the simplex search method (Lagarias et al., 1998)—similarly to the one used for the saccade model—that minimized the area between the original head data and the outcome of the model.

The gain *g*^*w*^ and the corresponding phase ϕ^*w*^ = δ*t*ω describe the relation between head and platform in world coordinates. Since we want to understand how the pigeon responds to the perturbation it experiences, we transformed these values into gain and phase of the system's response. The resulting response gain describes the response of the pigeon in a coordinate system that is fixed to the platform. The transformation from movement gains to response gains is based on:

(4) $$\begin{array}{r} x_{h}^{p}=x_{h}^{w}-x_{p}^{w} \end{array}$$

with the superscript indicating the coordinate system (world or platform coordinates).

Substituting $x_{p}^{w}=a_{p}\text{sin}(\omega t)$ and $x_{h}^{w}=g^{w}a_{p}\sin\left(\omega t-\phi^{w}\right)$ and using trigonometric arithmetic, it follows that:

(5) $$\begin{array}{r} x_{h}^{p}=a_{p}\sin\left(\omega t-\arctan\left(\frac{\sin\phi^{w}}{1-\cos\phi^{w}}\right)\right)\sqrt{g^{w2}-2g^{w}\cos\phi^{w}+1} \end{array}$$

Therefore, response gain and phase in platform coordinates can be described as:

(6) $$\begin{array}{r} g^{p}=\sqrt{g^{w2}-2g^{w}\cos\phi^{w}+1}\;and\;\phi^{p}=\arctan\left(\frac{\sin\phi^{w}}{1-\cos\phi^{w}}\right) \end{array}$$

### Statistical analysis

We used the Mann-Whitney U-Test to compare medians of gains between light and dark conditions, because data were not normally distributed. Phase differences were analyzed with the Watson-Williams-test as an equivalent of the Mann-Whitney U-Test for circular statistics (Berens, 2009). The comparison of active head saccades was analyzed with an ANOVA.

## Results

Head stabilization depended on the type of movement of the motion platform. It differed between translations and rotations, and it depended on whether or not visual flow information was available.

**Figure 2:**
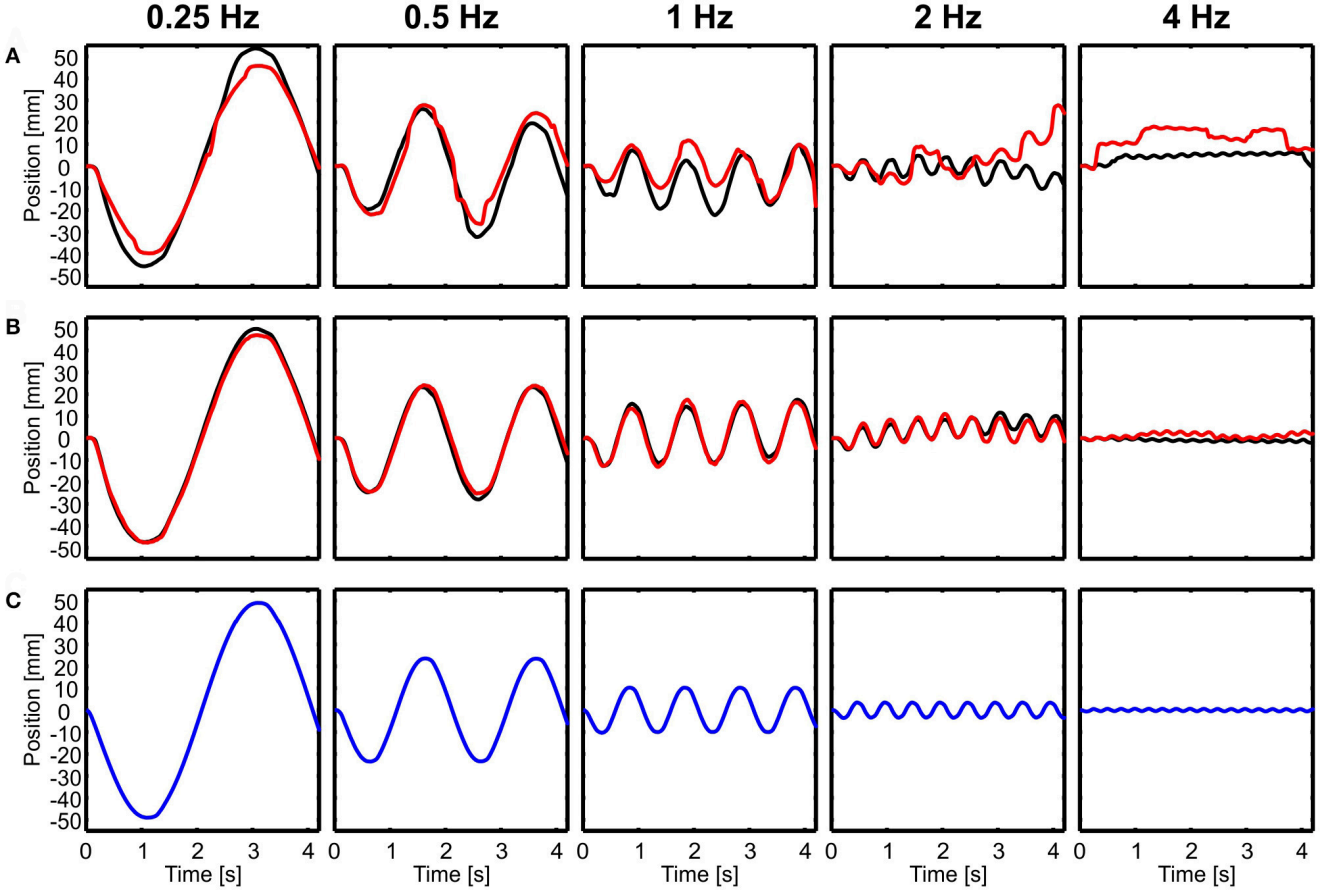
Examples of head and body movement in response to translational perturbations. Head **(A)** and body **(B)** positions in x-direction (forward-backward) are shown over time for five different frequencies (0.25–4 Hz) in light (red) and dark (black) conditions in response to the movement of the platform (**C**, blue). Note that both, the head and the body followed the movement of the platform.

During translations, body and head movements were similar to the movement of the platform, as shown by representative time courses in forward-backward direction (Figure 2). No compensation was observed and the head was not stabilized. This seemed to be frequency independent and even at 4 Hz, both, the body and the head moved with about the same amplitude as the platform. Small differences were observed between light and dark conditions for head motion (Figure 2A), but not for body motion (Figure 2B). Amplitude of head motion in light condition seemed to be slightly smaller than the amplitude of the platform movement.

In the example of Figure 2, it is also obvious that both head and body were lagging behind the platform movement for higher frequencies. A lag of 100 ms, which is about the amount observed in the example in the 4 Hz condition, translates into a considerable phase lag of 145 deg.

**Figure 3:**
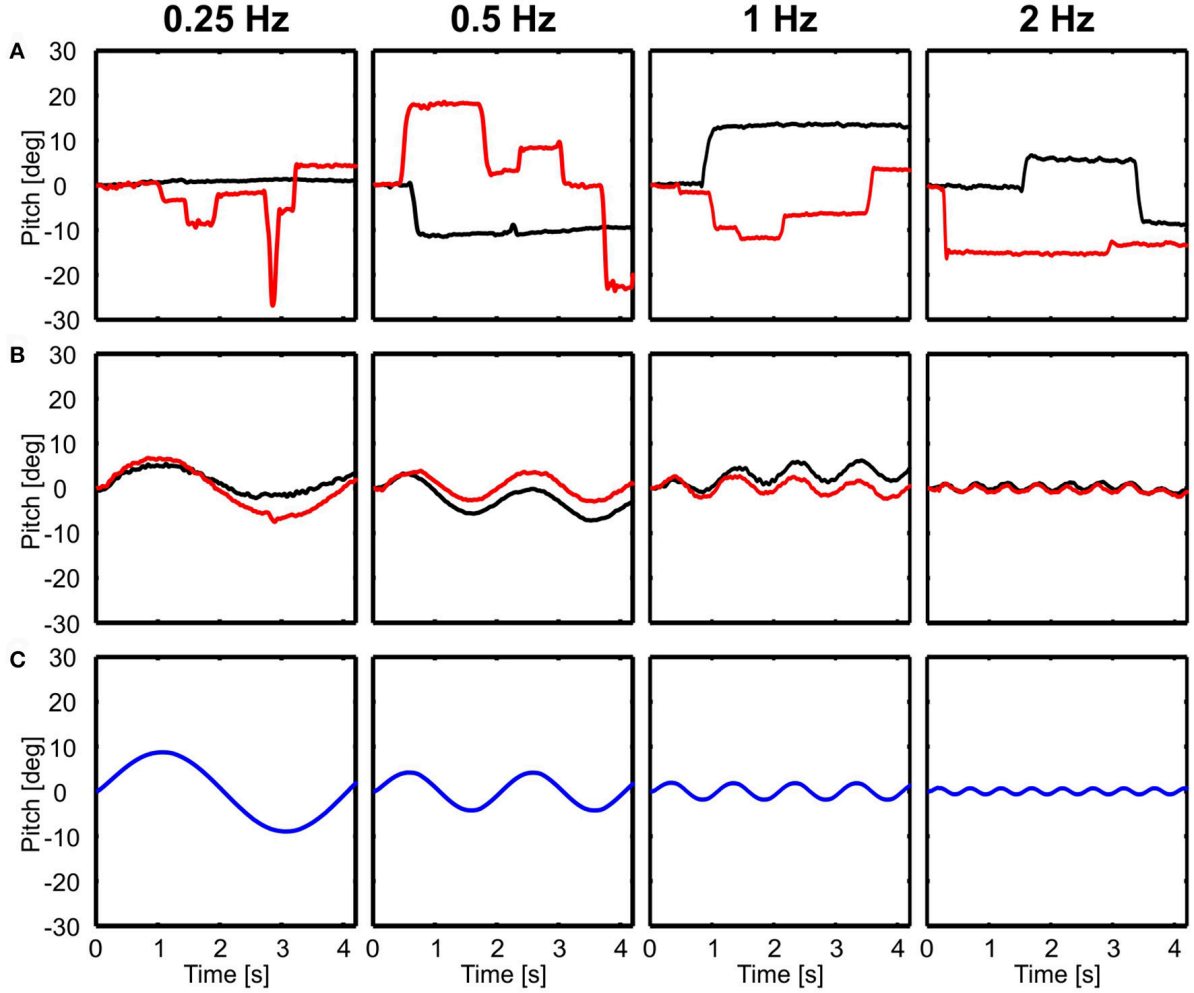
Examples of head and body movement in response to rotational perturbations. Head **(A)** and body **(B)** y-angles (rotation around the lateral axis) are shown over time for four different frequencies (0.25–2 Hz) in light (red) and dark (black) conditions in response to the movement of the platform (**C**, blue). Note that except from active head saccades the head was always stabilized.

In contrast to translational movements, only the body followed rotations (Figure 3B), whereas the head seemed to be perfectly stabilized aside from active rotational head saccades (Figure 3A). Here, examples of rotations around the lateral axis are shown and the head was always stabilized independent of light conditions (Figure 3).

### Poor head stabilization in response to translational perturbations

**Figure 4:**
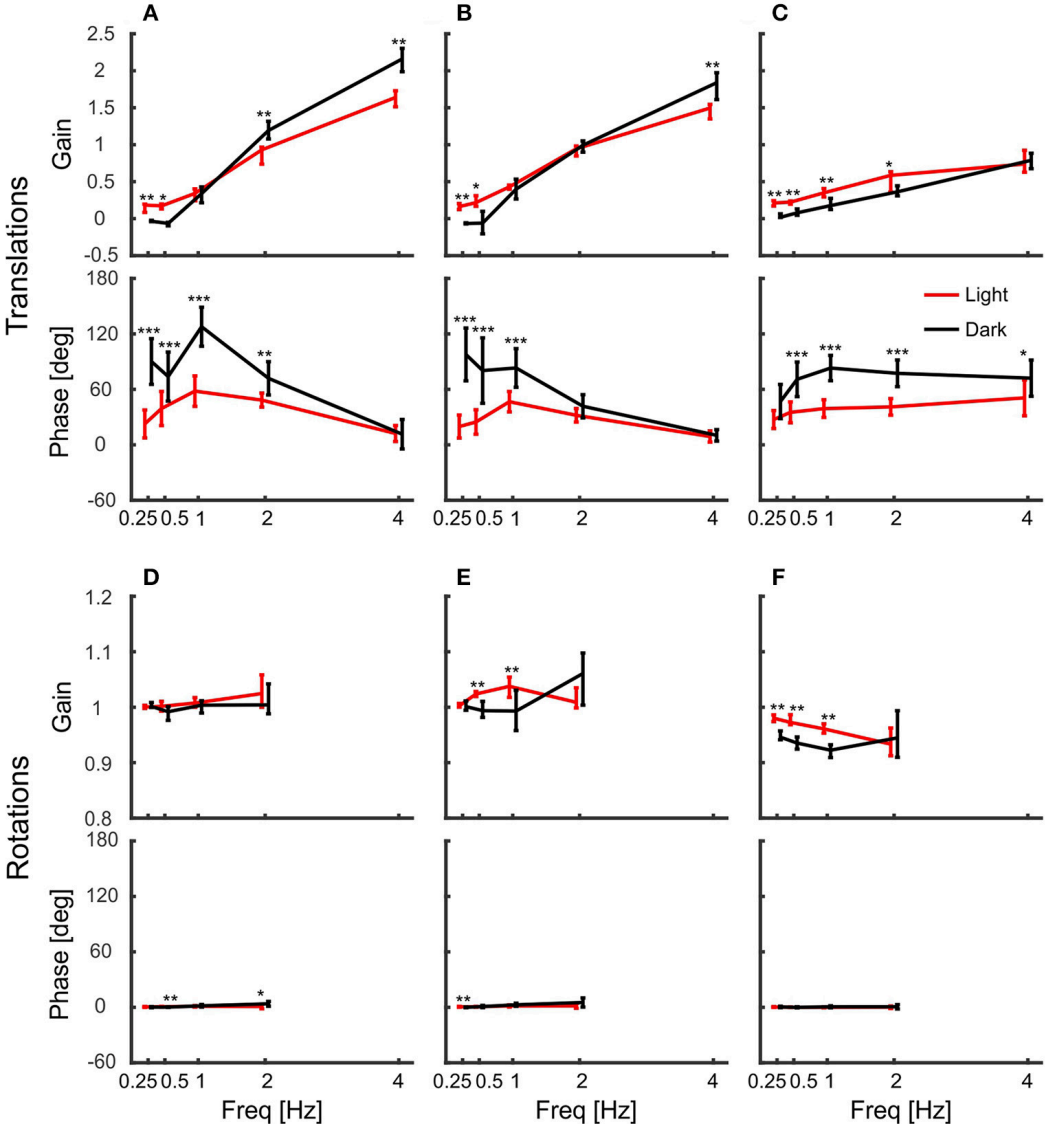
Pigeon actively compensate platform rotations but not translations with their head. **(A–C)** Median gains and mean phase shifts of the head in response to platform translations along the x-axis **(A)**, the y-axis **(B)**, and the z-axis **(C)**. **(D–F)** Gains and phases for the respective rotations around the x-axis **(D)**, the y-axis **(E)**, and the z-axis **(F)**. **(A–F)** Data are shown for light (red) and dark (black) conditions against frequency. A gain of one with a phase of zero indicates that the head was perfectly stabilized, whereas response gain of zero with phase zero means that the head moved with the same amplitude as the platform. Please note the different scales between translations and rotations. Error bars show 25 and 75 percentiles of the median gains and standard deviations of the mean phases of 10 animals. Asterisks indicate significant differences between light and dark conditions (*U*-Test, Watson-Willliams test, ^*^p < 0.05; ^**^p < 0.01; ^***^p < 0.001).

Figure 4 shows summary plots of the gains and corresponding phases of the head in response to the six degrees of platform perturbations for different frequencies and the two lighting conditions. Recall, that perfect head stabilization would mean a gain of one and a phase of zero. A gain of zero and phase of zero means that the head moves along with the platform. The head would also move with the same amplitude as the platform with a gain of one and a phase of 60 degrees, but it would lag behind the platform. Accordingly, with a gain of one and a phase of 180 degrees the amplitude of the head in world coordinates would be twice as large as that of the platform. From the data plotted in Figure 4, it becomes clear that the pigeons did not compensate translations very well. For low frequencies, gains were close to zero. With 2 Hz perturbations, gains approached a value of one, but phases were in the order of 60 degrees which means that head stabilization was not achieved either (Figures 4A,B; 2 Hz). At 4 Hz, gains became much larger than one, which again demonstrates a lack of stabilization. The increase of gain with frequency was smaller for vertical than for horizontal perturbations (compare Figure 4C with Figures 4A,B). For low frequencies, phase shifts differed between light and dark condition during translations (Figures 4A–C). However, in all cases, head stabilization was virtually absent.

### Accurate head stabilization in response to rotational perturbation

In contrast to the observations under translational perturbation, the head was almost perfectly stabilized when the platform was rotated. In principle, this was the case for all frequencies and for both light conditions (Figures 4D–F). During rotations, all gains were close to one and the phases were close to zero, indicating an active stabilization of the head. For rotations around x and y, the response gains were independent of the frequency, whereas there was very little decrease in gain for rotations around z for higher frequencies (Figure 4F).

**Figure 5:**
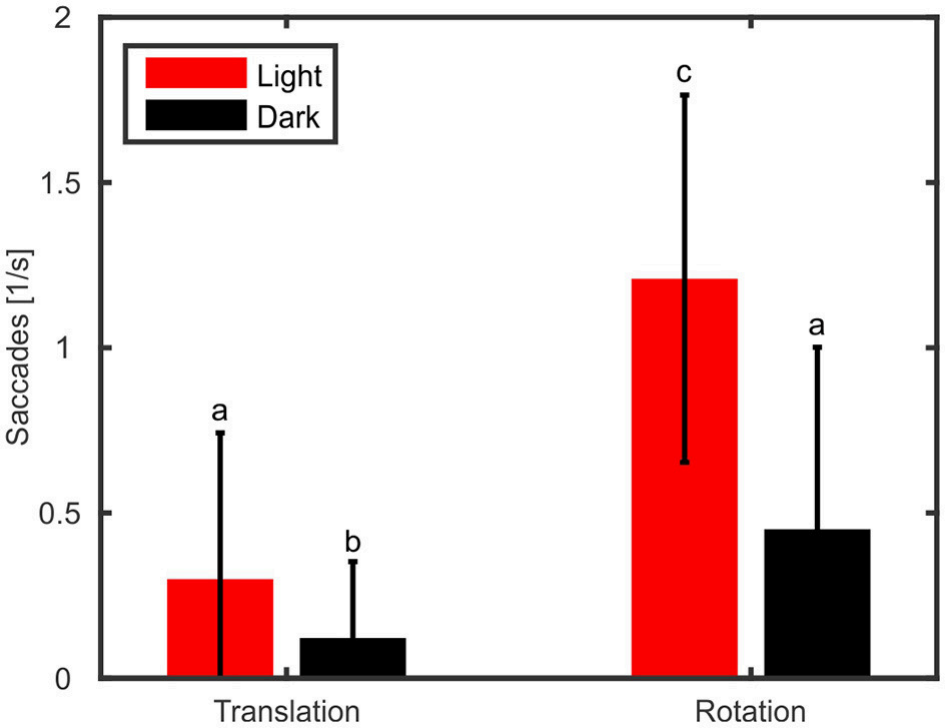
Rotation of the platform causes more active head saccades than translations. Mean numbers of head saccades per second and standard deviations are shown for translations and rotations during light (red) and dark (black) conditions. Letters indicate significantly different amounts (*p* < 0.05) of saccades (multiple comparison test following an ANOVA).

In addition to gain and phase differences, we evaluated the amount of head saccades per second (Figure 5) with a two-way ANOVA using type of movement (rotation vs. translation) and light condition (light on vs. darkness) as factors. The ANOVA revealed that type of movement had a significant effect on the amount of head saccades [ANOVA, *F*_(1, 36)_ = 135.5, *p* < 0.001]. Furthermore, head saccades were also significantly affected by light condition with fewer saccades observed in the dark [ANOVA, *F*_(1, 36)_ = 64.0, *p* < 0.001] and we found a significant interaction between light condition and type of movement [ANOVA, *F*_(1, 36)_ = 40.46, *p* < 0.001]. In the light condition, rotation of the platform resulted on average in 1.1 head saccades/s, which is about ten times more than during translations (0.1 saccades/s; Figure 5, red). This trend could also be observed in darkness (rotation: 0.3 saccades/s; translation: 0.03 saccades/s).

### Contributions of body and head

**Figure 6:**
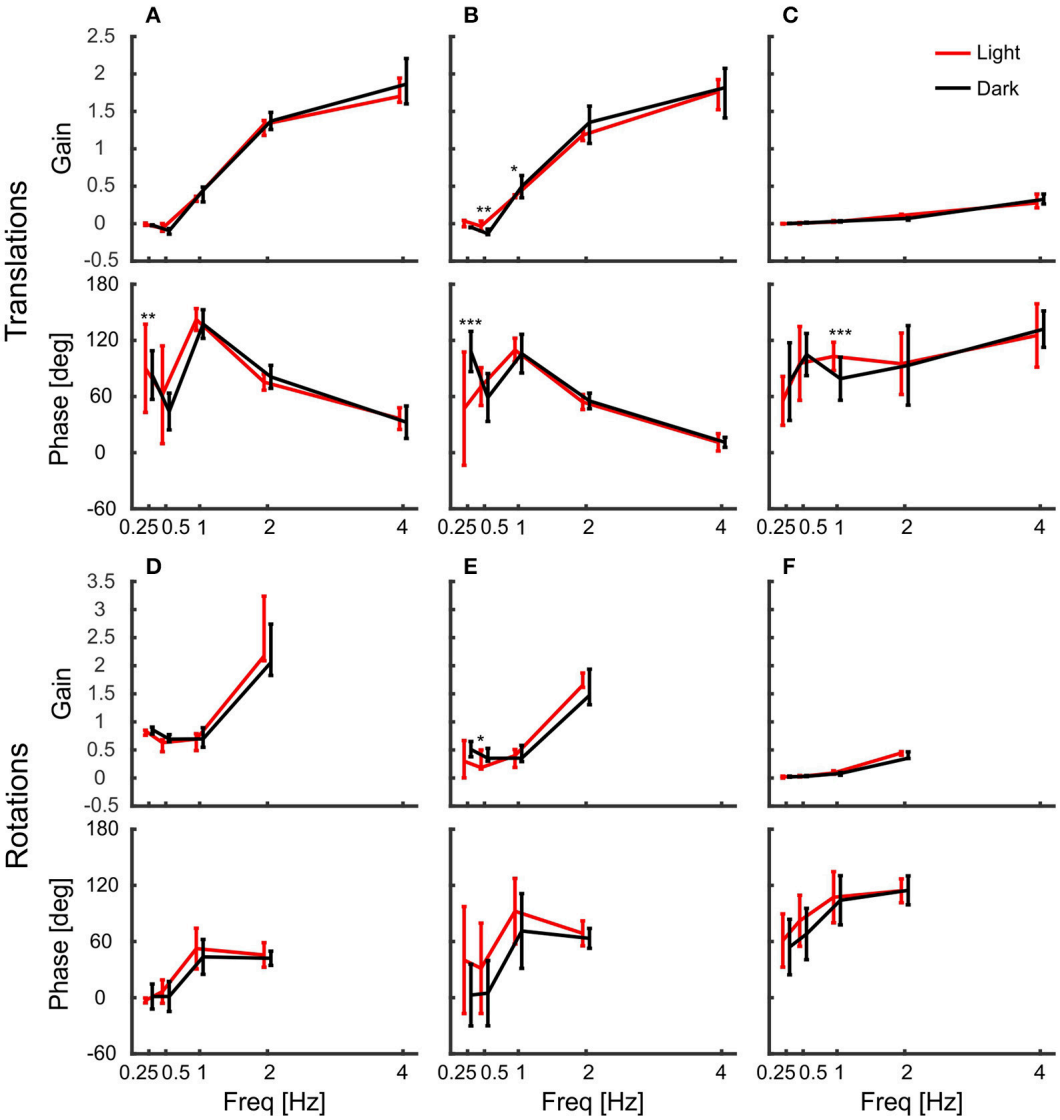
The body compensated low frequency/large amplitude x and y rotations. **(A–F)** Median gains and phases of the body in response to platform translations along the x-axis **(A)**, the y-axis **(B)**, and the z-axis **(C)**, and respective platform rotations around x **(D)**, y **(E)**, and z **(F)** are shown for light (red) and dark (black) conditions against frequency. All error bars show 25 and 75 percentiles of the median gains and standard deviation of the mean phase of 10 animals. Asterisks indicate significant differences between light and dark conditions (*U*-Test, Watson-Willliams test, ^*^p < 0.05; ^**^p < 0.01; ^***^p < 0.001).

The gains of the head showed that it was stabilized better during platform rotations than during translations. But how do the pigeons stabilize it? As the birds were freely standing on a perch, they could counteract platform movements not only with their neck, but also with their legs. Therefore, we also tracked the back of the pigeons and calculated the gain of the body with respect to the platform movement (Figure 6). The results show that the body was not stabilized against platform translations (Figures 6A–C). Instead, body gain was close to zero for low frequencies and increased above one with increasing frequency in x- and y-directions (Figures 6A,B). This indicates that the body moved even further than the platform for high frequencies. In the vertical direction, body gain was always close to zero independent of frequency indicating that the pigeons did not compensate vertical movements with their legs (Figure 6C).

Similarly, they did not counteract rotations around the vertical axis, for which body gain was also close to zero (Figure 6F). This was different for the other rotations. Pigeons compensated rotations around x and y when the platform moved with low frequencies and high amplitudes (Figures 6D,E). At the lowest frequency, the median gain for rotation around x was 0.86 in darkness. This means that the average movement amplitude of the body was 1.4 degree, when the platform was rotated about 9 degrees. Interestingly, the movement amplitude of the body remained similar during rotations around x with higher frequencies (1.6, 1.5, and 1.9 degree, for 0.5, 1, and 2 Hz respectively).

**Figure 7:**
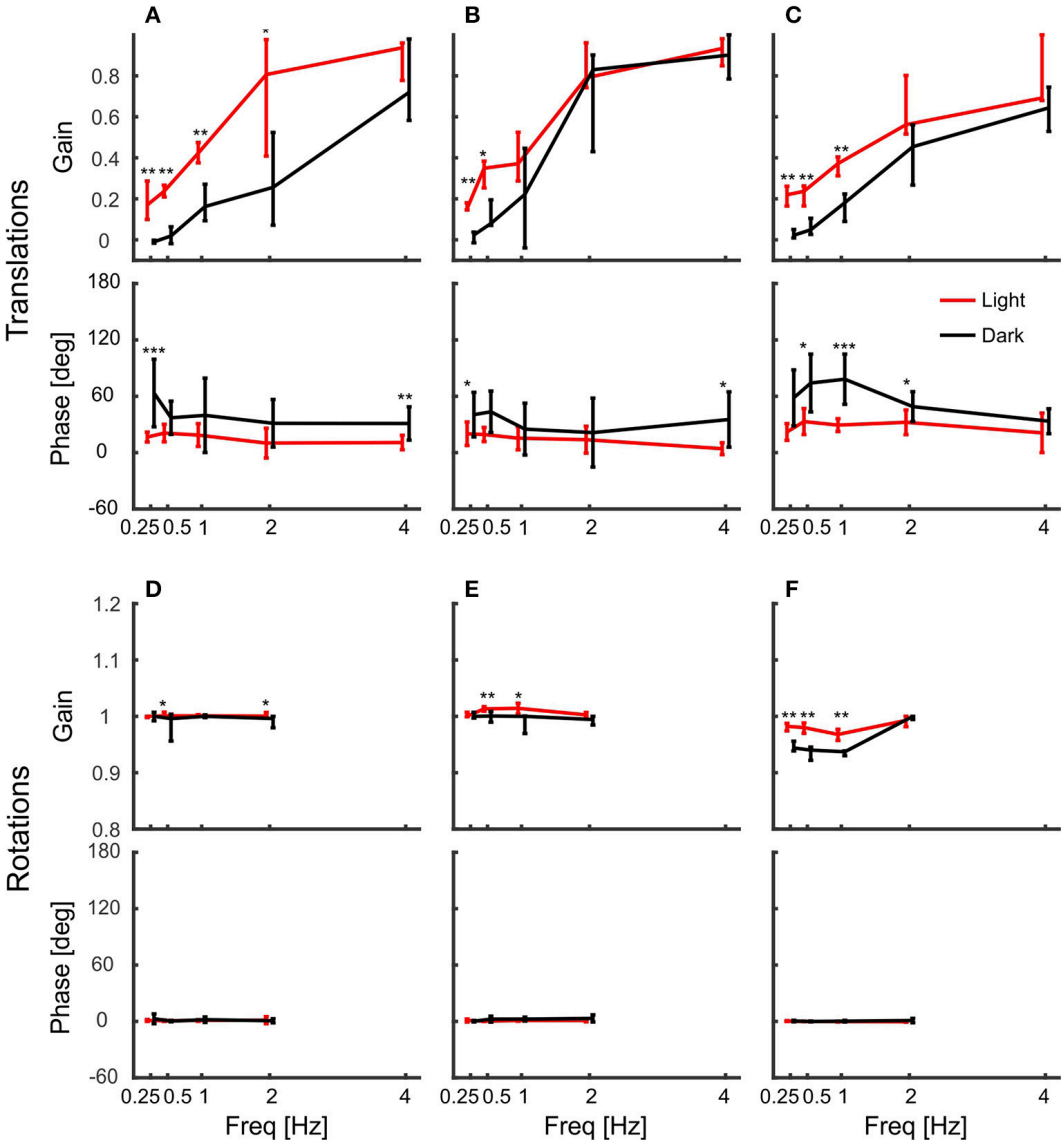
Head gain with respect to body movement indicates perfect head stabilization. **(A–F)** Median gains and phases of the head in response to body translations along the x-axis **(A)**, the y-axis **(B)**, and the z-axis **(C)**, and respective body rotations around x **(D)**, y **(E)**, and z **(F)** are shown for light (red) and dark (black) conditions against frequency. Error bars show 25 and 75 percentiles of the median gains and standard deviations of mean phases of 10 animals. Asterisks indicate significant differences between light and dark conditions (*U*-Test, Watson-Willliams test, ^*^p < 0.05; ^**^p < 0.01; ^***^p < 0.001).

Body movement obviously affects head position, which is why we also calculated gain and phase of the head with respect to the body (Figure 7). In contrast to head gains with respect to platform movement (Figure 4), gains with respect to body movement remained below one during translations and the corresponding phases were less variable (Figures 7A–C). For low frequencies, gains were significantly higher in light than in darkness, indicating better head stabilization in light conditions. Nevertheless, the head was not much stabilized against body translations. In contrast, gains and phases indicated a perfect head stabilization during rotations (Figures 7D–F). Although rotations around z were less stabilized than rotations around x and y, all response gains were close to one and the phases always close to zero with little variability.

## Discussion

Our results show that pigeons' head stabilization differs between translations and rotations. In contrast to translations, where the head largely followed the movement of the motion platform, the head was almost perfectly stabilized during rotations. The results also show that vision has a small but measurable effect on head stabilization during translation, and that, in contrast, visual information is clearly not necessary to stabilize the head during rotations.

The latter finding contradicts the assumption that head stabilization is mainly based on visual feedback as suggested by earlier studies (Friedman, 1975; Frost, 1978). These authors showed that visual stimulations are sufficient to induce head-bobbing and concluded that head stabilization is based on an optokinetic response, because head-bobbing includes a stable hold phase. Similar to our results of head stabilization during translations, these optokinetic responses are frequency dependent and show an increase in gain and phase lag with increasing frequency of optical stimuli (Wallman and Velez, 1985; Gioanni, 1988a). One possible reason for the lack of head stabilization during translation and for the small effects of visual feedback in our experiments could be that the cage around the animal as well as the platform itself moved along with the pigeon. Specifically the lower part of the visual field does not provide the optic flow required for head stabilization. If visual head stabilization is driven mainly by the ground, then we would not expect to see head stabilization in our experiment.

Very good stabilization, however, is clearly present in response to rotational perturbations, both under light and under dark conditions. This clearly indicates that vision is not necessary and that the very precise control of the head under rotational perturbation is based on other sensory modalities. Vestibular information could be used in principle, but as mentioned above, only within a feedback controller, which is inherently slow and likely to generate observable control errors. The outstanding precision of head control during rotational perturbations thus points to other sensory systems that can directly measure changes in orientation that, in turn, can be used as input for faster feed-forward control.

In pigeons, the eyes move with the head and there are only little eye movements within the head (Bloch et al., 1988; Lemeignan et al., 1992). Therefore, head stabilization serves the stabilization of the retinal image, which explains the fact that the head is differently stabilized during translations and rotations. Translations lead to optic flow that contains important self-motion and distance information about objects in the environment. In contrast, image rotations do not contain such useful information. Therefore, it makes sense that the head is stabilized only partly against translations, but that rotations are fully compensated. This is in accordance to the finding that pigeons, if necessary at all, only compensate head rotations, but not translations with eye movements based on the VOR (Dickman and Angelaki, 1999). However, the VOR is under-compensatory for head rotations (Dickman et al., 2000) and in a head-fixed condition the gain was only about 0.46 when the body was moved (Haque and Dickman, 2005). If the head is free to move, the majority of the stabilization of the retinal image is done by the head, which accounts for about 80% of the gaze stabilization (Gioanni, 1988b; Haque and Dickman, 2005).

The VOR and the VCR are based on feedback from the vestibular system, but it would not make much sense if vestibular feedback would be used to stabilize the body, because the head moves with the body. In addition to proprioceptive feedback from the legs, the lumbosacral system in the spine might provide feedback about the body orientation (Singer, 1884; Necker et al., 2000). Necker (2005) suggested that the lumbosacral system provides an equilibrium sense similarly as the vestibular system and that it provides sensory feedback about rotational movements. In an experiment where the spine was cut, pigeons still showed reflexes such as lifting their tail and wings in response to rotations (Singer, 1884). Furthermore, after lesions of these lumbosacral anatomical specializations, pigeons had problems to keep balance on a rotating perch (Necker et al., 2000). Especially during walking the lumbosacral system serves stability (Necker, 2006), which indicates that it might also play an important role for balance and head stabilization during standing. While the functionality of the lumbosacral vertebral canal system remains speculative, it is certainly a candidate for providing information about changes of body orientation needed for fast feed-forward control of the head.

The vestibulo-colic reflex (VCR), like the optokinetic response, also depends on frequency. In response to yaw rotations, head stabilization improved with higher frequencies in both, light and dark conditions (Gioanni, 1988b). However, even in light conditions the head was not perfectly stabilized, but gains increased from 0.8 for 0.2 Hz to about 0.9 at 1 Hz (Gioanni, 1988b). This was different in our experiment, where head stabilization decreased with an increasing frequency during rotations around z, but was always near to perfect in response to any rotation. It is unlikely that these differences only result from methodological differences, such as stimulation amplitude, frequency, velocity and acceleration, but instead, one major difference was that in our experiments the birds stood free compared to restrained sitting birds. On the one hand, head stabilization might be influenced by the different states (free standing vs. restrained sitting), because it was shown to be state dependent: pigeons that are in a “flying” mode stabilize their head better than hanging birds (McArthur and Dickman, 2011). On the other hand, free standing allows to compensate rotations with the body. However, our results show that only rotations around x and y were partly compensated by the body and that the body did not contribute to head stabilization during rotations around the vertical axis (z). This makes sense, because only rotations around x and y cause instability and falls if the pigeon does not counteract the rotation appropriately. However, the almost perfect head stabilization with respect to body movement (Figure 7) indicates that body movement alone does not explain the differences in head stabilization between unrestrained and fixated pigeons. Another major factor of unrestrained standing is that it provides additional proprioceptive sensory feedback from the feet and legs. This proprioceptive feedback from the legs might be integrated with vestibular and visual feedback and therefore might contribute significantly to head stabilization.

## Conclusions

We found that head stabilization is almost perfect when the pigeons are standing free on a rotating perch. Visual feedback is likely too slow to account for this accuracy. Vestibular information is generated and transmitted faster, but it can only be used within a feedback controller and not within a faster feed-forward mechanism, which is likely required to achieve the high precision of control that we observed. Therefore, we suggest that head stabilization in pigeons is not only based on vestibular and visual feedback, but consider it likely that proprioception and additional sensory systems like the lumbosacral system in the spine provide information for a feed-forward mechanisms. Further experiments should be conducted to investigate the contribution of proprioceptive and other potential body-centered sensory systems.

## Author contributions

LT designed experiments, acquired and analyzed data, interpreted the findings and wrote and revised the manuscript. NT designed experiments, supervised LT, interpreted findings and edited the manuscript.

### Conflict of interest statement

The authors declare that the research was conducted in the absence of any commercial or financial relationships that could be construed as a potential conflict of interest.
