# Supplementary material for: Head Stabilization in the Pigeon: Role of Vision to Correct for Translational and Rotational Disturbances
Source: Front Neurosci. 2017 Oct 5;11:551. doi: 10.3389/fnins.2017.00551 (PMC5633612; doi:10.3389/fnins.2017.00551)
Supplement: Supplementary file 1 [file Table1.DOCX]

Supplementary Table 1: Average maximum amplitudes, velocities and accelerations of the motion platform for all frequencies and directions (translations and rotations: X, Y, Z).

| Translations | X | | | Y | | | Z | | |
| --- | --- | --- | --- | --- | --- | --- | --- | --- | --- |
| [Hz] | [mm] | [mm/s] | [mm/s^2^] | [mm] | [mm/s] | [mm/s^2^] | [mm] | [mm/s] | [mm/s^2^] |
| 0.25 | 49.7 | 80.0 | 624.5 | 49.6 | 79.3 | 610.6 | 49.2 | 79.8 | 887.3 |
| 0.5 | 23.7 | 76.7 | 637.4 | 23.6 | 75.9 | 590.5 | 24.7 | 75.3 | 911.8 |
| 1 | 10.5 | 70.8 | 884.4 | 10.4 | 71.8 | 818.2 | 10.7 | 66.2 | 892.1 |
| 2 | 3.8 | 53.7 | 867.3 | 3.6 | 49.6 | 1050.8 | 4.4 | 48.2 | 842.7 |
| 4 | 0.8 | 15.1 | 486.6 | 0.7 | 15.3 | 423.7 | 1.1 | 21.7 | 613.9 |
|  | | | | | | | | | |
| Rotations | X | | | Y | | | Z | | |
| [Hz] | [deg] | [deg/s] | [deg/s^2^] | [deg] | [deg/s] | [deg/s^2^] | [deg] | [deg/s] | [deg/s^2^] |
| 0.25 | 9.1 | 15.2 | 133.0 | 8.9 | 15.3 | 197.7 | 9.0 | 14.4 | 94.7 |
| 0.5 | 4.4 | 13.9 | 116.3 | 4.4 | 14.4 | 196.2 | 4.3 | 13.6 | 109.9 |
| 1 | 1.9 | 12.3 | 131.2 | 2.0 | 12.7 | 182.8 | 1.8 | 12.3 | 143.2 |
| 2 | 0.9 | 9.3 | 192.0 | 0.8 | 9.5 | 174.0 | 0.7 | 9.5 | 141.3 |
